# Curcumin and Baicalin Co-Loaded Nanoliposomes for Synergistic Treatment of Non-Small Cell Lung Cancer

**DOI:** 10.3390/pharmaceutics16080973

**Published:** 2024-07-23

**Authors:** Qijun Su, Junqian Pan, Chunxin Wang, Meng Zhang, Haixin Cui, Xiang Zhao

**Affiliations:** Institute of Environment and Sustainable Development in Agriculture, Chinese Academy of Agricultural Sciences, Beijing 100081, China; 82101215249@caas.cn (Q.S.); panjunqian@caas.cn (J.P.); wangchunxin@caas.cn (C.W.); 82101225293@caas.cn (M.Z.); cuihaixin@caas.cn (H.C.)

**Keywords:** nanoliposomes, curcumin, baicalin, non-small cell lung cancer, nano-delivery system

## Abstract

Currently, the treatment of patients with advanced non-small cell lung cancer (NSCLC) mainly relies on traditional chemotherapeutic drugs; however, most of them have limited therapeutic effects and high toxicity. Some natural products with good therapeutic efficacy and low toxicity and side effects are limited in clinical application due to their low solubility and bioavailability. In this study, a nanoliposome drug-carrying system (Lip-Cur/Ba) was developed for the co-delivery of curcumin (Cur) and baicalin (Ba) using the thin-film hydration method. In vitro experiments demonstrated that Lip-Cur/Ba had a strong killing effect on A549 cells, and the inhibitory effect of Lip-Cur/Ba on A549 cells was enhanced by 67.8% and 51.9% relative to that of the single-carrier system, which could reduce the use of a single-drug dose (Lip-Cur and Lip-Ba), delay the release rate of the drug and improve the bioavailability. In vivo experiments demonstrated the antitumor activity of Lip-Cur/Ba by intravitreal injection in BALB/c mice, and there were no obvious toxic side effects. This study provides a new idea for curcumin and baicalin to be used in the co-treatment of NSCLC by constructing a new vector.

## 1. Introduction

Lung cancer is a common type of cancer that has become the number one contributor to malignancy deaths worldwide [1]. Non-small cell lung cancer (NSCLC) is a common classification of lung cancer, accounting for approximately 80% of cases [2]. In addition, about 35% of patients suffering from NSCLC are already in intermediate to advanced stages at the initial diagnosis, while the 5-year survival rate of patients with stage IV is only 5.8% [3]. The current standard of care for early-stage NSCLC patients is surgical resection, which can achieve some therapeutic effect [4]. However, most of the patients in the middle and late stages of NSCLC are unable to reach the standard of surgical resection due to poor health and other factors, and they can only improve their condition and maintain their survival through chemotherapy and radiation therapy [5]. Nevertheless, due to the high toxicity and side effects of traditional treatments, such as nausea and vomiting, liver function damage, decreased immunity and other symptoms [6], as well as the limited therapeutic effect, it is necessary to research and develop more new therapeutic methods to deal with NSCLC.

Curcumin (Cur) and baicalin (Ba) are both extracted from natural sources, and several studies have confirmed the beneficial effects of these two substances on the human body [7]. Cur is a polyphenol compound mainly extracted from the Curcuma root, which is widely used for its anticancer, antibacterial and antioxidant properties. In recent years, the anticancer activity of curcumin has been focused on lung cancer, liver cancer, cervical cancer and so on [8,9,10]. For example, it has been proved that the Cur anticancer effect on NSCLC is to inhibit the miR-21 signaling pathway to promote the elevation of the PTEN gene, thus inhibiting cell proliferation and promoting cancer cell apoptosis [11]. In addition, Cur inhibits the proliferation of lung cancer cell lines and induces apoptosis in A549 cells by affecting the Wnt/β protein signaling pathway [12]. Ba is a flavonoid extracted from the root of *Scutellaria baicalensis*, which has significant biological activities such as antibacterial, anti-inflammatory and anticancer effects [13,14,15]. Ba has been shown to inhibit liver cancer, breast cancer and lung cancer [16,17,18]. For example, Ba inhibits the growth of NSCLC cells by inhibiting PBK/TOPK and downstream signaling molecules histone H3 and ERK2 in vitro [19]. In addition, it has been demonstrated that Cur and Ba can be co-administered for the treatment of hepatitis in rats, and that their joint inhibition of the TSC1/eIF-2α/ATF4 pathway synergizes in lung diseases [20]. There have also been studies on the co-encapsulation of Cur and Ba by nano-micelles as carriers for the treatment of NSCLC [21]. Therefore, the potential synergistic effect of Cur and Ba and their combination therapy has become an interesting research direction, but there are no studies on the co-encapsulation of Cur and Ba using nano-liposomes as a carrier, which may be due to the immature technology of the preparation of the liposomes, and the low solubility, bioavailability and poor stability of the drug.

Liposomes are lipid delivery systems made by encapsulating or embedding active ingredients in lipid-like nuclei as a closed vesicular substance similar to the structure of biological membranes, formed by encapsulating and using phospholipids and cholesterol as membrane materials [22]. Nanoliposome is a new type of lipid nanocarrier developed on the basis of liposomes, with particle sizes between 10 and 1000 nm. Nanoliposomes can improve the solubility and bioavailability of difficult-to-solve drugs and enhance the efficacy of drugs to enhance their absorption by the human body [23]. To date, an increasing number of FDA-approved liposome-based biologics and clinical therapeutics have been developed in a wide range of fields, including anticancer, antimicrobial, etc. [24].

In this study, we developed a nanoliposome delivery system (Lip-Cur/Ba) co-encapsulating curcumin and baicalin; characterized the morphology, size and structure of Lip-Cur/Ba; demonstrated the successful encapsulation of the drugs in liposomes; evaluated their in vitro release and in vivo antitumor activity; and proved the synergistic inhibitory effect of Lip-Cur/Ba on A549 cells by in vitro experiments and the antitumor activity by intravitreal injection in BALB/c mice with no obvious toxic side effects.

## 2. Materials and Methods

### 2.1. Materials

Curcumin and baicalin were purchased from Baoji Chenguang Biological Co., Ltd. (Baoji, China); egg yolk lecithin (EYL) was purchased from Shanghai Yuanye Biotechnology Co., Ltd. (Shanghai, China); cholesterol (Chol) was purchased from Solebaum Biotechnology Co., Ltd. (Beijing, China); methanol and chloroform were purchased from McLean Biochemical Co., Ltd. (Shanghai, China); Dulbecco’s minimum essential medium (DMEM) was obtained from Pricella Biotechnology Co., Ltd. (Shanghai, China); fetal bovine serum (FBS) was obtained from Thermo Fisher Scientific Co., Ltd. (Shanghai, China); the A549 cell line was purchased from Punosai Life Science and Technology Co., Ltd. (Wuhan, China); the ROS assay kit was purchased from Beyotime Biological Co., Ltd. (Shanghai, China); and the BALB/c mice (male, 5 weeks old) were purchased from Guosheng Zhongyuan Science and Technology Company (Tianjin, China) under the license no. SCXK(Beijing)2019-0008. All the animal experiments complied with the guidelines of the Tianjin Medical Experimental Animal Care, and animal protocols were approved by the Institutional Animal Care and Use Committee of Yi Shengyuan Gene Technology (Tianjin) Co., Ltd. (protocol number YSY-DWLL-2023251).

### 2.2. Methods

#### 2.2.1. Preparation of Lip-Cur/Ba, Lip-Cur, Lip-Ba and Blank Liposomes (B-Lip)

Lip-Cur/Ba was prepared using a thin-film hydration-ultrasonic method [25,26,27]. EYL (140 mg), Chol (20 mg), Cur (5 mg) and Ba (6 mg) were dissolved in a mixture of methanol (6 mL) and chloroform (6 mL) and stirred until complete dissolution. The mixture was then added to a round-bottomed flask and all the solvent was evaporated at 42 °C using a rotary evaporator to form a homogeneous film inside the flask wall. Then, 10 mL of PBS solution preheated to 40 °C was added so that the film was completely dissolved in the PBS solution and hydrated to obtain the liposome suspension. Next, the liposome suspension was magnetically stirred at 40 °C for 1 h. This was to make the liposome suspension more homogeneous and completely dissolved. Finally, the liposome suspension was sonicated using a cell crusher for 10 min to obtain an aqueous solution of Lip-Cur/Ba. Blank-Lip(B-Lip) without drugs and single-drug loaded Lip-Cur and Lip-Ba were prepared according to the same method.

#### 2.2.2. Encapsulation Efficiency and Loading Capacity Determination of Lip-Cur/Ba

The encapsulation efficiency and loading capacity were determined using UV spectrophotometry. The Lip-Cur/Ba was centrifuged at 10,000 r/min for 5 min at a high speed. At this time, the drugs not encapsulated into the liposome were free in the water to form crystals precipitation, the high-speed centrifugation aggregated these drugs into the precipitate [28], the supernatant was taken and methanol was added to completely dissolve the liposomes and the encapsulated drug, the contents of Cur and Ba were determined using an ultraviolet spectrophotometer (Shimadzu, UV2600) at 425 nm and 278 nm [29,30] and Lip-Cur and Lip-Ba were determined using the same method. In addition, the encapsulation efficiency (EE) and the loading capacity (LC) were calculated according to the following equation:

$$EE(\%)=\frac{W_{1}+W_{2}}{W_{d}}\;\times \;100\%$$

$$LC(\%)=\frac{W_{1}+W_{2}}{W_{m}}\;\times \;100\%$$

where W_1_ and W_2_ are the mass of Cur and Ba embedded in the liposome, W_d_ is the total amount of both drugs added to the liposome and W_m_ is the total mass of the entire drug-loaded liposome.

#### 2.2.3. Characterization of Lip-Cur/Ba

The morphology of Lip-Cur/Ba was observed using transmission electron microscopy. The Lip-Cur/Ba solution was diluted 5 times, held in a test tube using an ultrasonic cleaner to treat the sample at a low power for 15 min and then dripped onto a copper mesh, allowed to dry and then mounted on a machine and observed by imaging at 80 kV.

#### 2.2.4. Size and Zeta Potential

The particle size, PDI and zeta potential of Lip-Cur/Ba were determined using a laser particle sizer. The appropriate amount of the Lip-Cur/Ba solution was diluted and added into the cuvette then placed into the instrument, the temperature was set at 25 °C and the determination was repeated three times to take the average value.

#### 2.2.5. Fourier-Transform Infrared (FT-IR) Spectroscopy

Infrared spectral analysis of B-Lip, Cur, Ba and Lip-Cur/Ba was performed using a Fourier-transform infrared (FT-IR) spectrometer. The liposome samples of B-Lip and Lip-Cur/Ba were lyophilized to a powder form. The samples were taken separately and mixed with an appropriate amount of KBr and pressed into tablets using a tablet press. The tablets were then examined on the machine and scanned in the range of 400–4000 cm^−1^.

#### 2.2.6. Thermogravimetric Analysis

The weight losses of B-Lip, Cur, Ba and Lip-Cur/Ba were determined using a thermogravimetric analyzer. The samples of B-Lip and Lip-Cur/Ba were lyophilized to a dry powder form. The samples were heated from room temperature to 800 °C in nitrogen at a rate of 20 °C/min to test the heating curves.

#### 2.2.7. Differential Scanning Calorimetry (DSC) Analysis

Thermal analyses of B-Lip, Cur, Ba and Lip-Cur/Ba were carried out using a differential scanning calorimeter. Samples (5 mg) were separately packed in pure aluminum crucibles and analyzed by heating from room temperature to 200 °C at 10 °C/min in nitrogen gas and later cooling down to 20 °C [27].

#### 2.2.8. In Vitro Releasing Assay of Lip-Cur/Ba

In vitro release kinetic studies were carried out using dialysis. The release medium was PBS solution containing 20% ethanol. A solution of 1 mL of Lip-Cur/Ba was placed into a dialysis bag (10 KDa), tied at both ends with string, placed into 80 mL of the release solution and placed in a shaker at a temperature of 37 °C at 130 rpm. The control was the same concentration of solution of Cur and Ba dissolved using methanol. At the 2 h, 4 h, 6 h, 8 h, 12 h, 24 h and 48 h of dialysis, 1 mL was taken from the release solution and then supplemented with 1 mL of fresh release solution [31]. The content of Cur and Ba in the removed release solution was determined using a UV spectrophotometer and the release rate was calculated.

#### 2.2.9. Cell Cytotoxicity Assays

MTT assay (3-(4,5-dimethyl-2-thiazolyl)-2, 5-diphenyl-2H-tetrazolium bromide) was used to evaluate the toxicity of Lip-Cur/Ba to A549 cells in vitro. B-Lip, Lip-Cur, Lip-Ba and Lip-Cur/Ba solutions were prepared separately, free Cur, Ba and Cur/Ba were dissolved in DMSO as controls and gradient dilutions were performed using DMEM culture medium. A549 cells in a logarithmic growth phase were selected and then digested with 1 mL of EDTA-containing trypsin. Then, the concentration of cell suspension was diluted to 1 × 10^5^ cells/mL, added to 96-well plates at 100 μL per well and 100 μL PBS was added in the peripheral-most wells to prevent evaporation and cultured until the cells were completely adherent to the wall. After wall attachment, the culture medium in the 96-well plate was discarded, the prepared culture medium containing the drug at different concentration gradients was added and each group was repeated for 6 wells, while the blank control group was added with culture medium without drugs. After a certain time of co-culture between the drug and the cells, the original culture medium in the wells was discarded and washed with PBS solution 2–3 times, 100 μL DMEM culture medium was reintroduced and then 10 μL MMTT solution was added to each well and continued to be incubated at 37 °C for 3 h. At the end of the incubation, the supernatant was aspirated out, 100 μL DMSO was added to each well and then the wells were shaken in the shaking table at 37 °C for 10 min, so as to completely dissolve the purple crystals. Finally, the absorbance was detected at 490 nm using an enzyme marker [32]. The cell viability was calculated according to the following formula:

$$Cell\;viability(\%)=\frac{OD\text{-}test}{OD\text{-}control}\;\times \;100\%$$

where OD-test is the absorbance of the drug addition experimental group and OD-control is the absorbance of the control group. The IC50 of Lip-Cur/Ba was calculated based on the cell viability and the CI of the combination of the two drugs was calculated according to the following equation:

$$CI=\frac{{\left(D\right)}_{1}}{{\left(D_{x}\right)}_{1}}+\frac{{\left(D\right)}_{2}}{{\left(D_{x}\right)}_{2}}$$

where (D_x_)_1_ and (D_x_)_2_ are the doses of Lip-Cur or Lip-Ba, respectively, required to achieve a given survival rate, and D_1_ and D_2_ are the doses of Cur and Ba, respectively, required to achieve the same survival rate when combined (Lip-Cur/Ba).

#### 2.2.10. Cell Scratch Assays

A cell scratch assay was used to evaluate the lateral antimigration ability of Lip-Cur/Ba for A549 cells. A549 cells were inoculated in six-well plates, and after all the cells had grown all over the whole plate bottom, the old culture medium was discarded and a serum-free culture medium was added to starve the cells for 24 h. A 20 μL lance tip was used to draw a line along the ruler on the bottom of the six-well plate, three lines were drawn in each well and then some PBS was added to clean the cells that had fallen out of the wells. Lip-Cur, Lip-Ba and Lip-Cur/Ba solutions were prepared separately and diluted to a concentration of 80 μg/mL using serum-free culture medium, and then added into the six-well plate to co-culture with the cells [33]. At 0 h and 48 h of culture, three fields of view were selected at the same position using an inverted microscope to photograph the scratches, and the area of the scratches was counted using Image J to calculate the migration rate. The migration rate was calculated according to the following formula:

$$Scratch\;healing\;rate(\%)=\frac{0\;h\;scratch\;areas-48\;h\;scratch\;areas}{0\;h\;scratch\;areas}\times 100\%$$

#### 2.2.11. Cellular Transwell Assays

A cellular Transwell assay was used to evaluate the longitudinal antimigration ability of Lip-Cur/Ba for A549 cells. A549 cells grown to a logarithmic phase were starved with serum-free culture medium for 24 h, digested with EDTA-free trypsin and then the cells were resuspended to a concentration of 5 × 10^4^ cells/mL using serum-free culture medium by adding 100 μL of cell suspension to the upper chamber of the Transwell and 1 mL of culture medium with a serum concentration of 20% to the lower chamber. At the same time, Lip-Cur, Lip-Ba and Lip-Cur/Ba solutions were prepared and added to the upper chamber so that the drug concentration in the upper chamber was finally 80 μg/mL, and then placed in the incubator for 48 h. At the end of the incubation, the culture solution in the upper and lower chambers of the Transwell were discarded, the upper chamber was washed with PBS three times and the migrated cells on the upper surface of the upper chamber were gently wiped away with a clean cotton swab. Then, an appropriate amount of methanol was added into the upper and lower chambers of the Transwell, respectively, and the cells were fixed for 20 min and washed with PBS three times after fixation. Finally, the Transwell upper chamber was dipped in 0.1% crystal violet dye solution for 20 min and washed with water to remove the excess dye [34]. After drying, the cell migration on the outer membrane of the upper chamber of the Transwell was observed and photographed under an inverted microscope to record the cell migration, and the number of cells migrated was counted by Image J. The cell migration rate was calculated according to the following formula:

$$migration\;rate(\%)=\frac{Number\;of\;cells\;migrating\;in\;the\;Lip\text{-}Cur/Ba}{Number\;of\;cells\;migrating\;in\;the\;Control}\times 100\%$$

#### 2.2.12. Detection of the Intracellular Reactive Oxygen Species (ROS) Level

The effect of Lip-Cur/Ba on the accumulation of reactive oxygen species in A549 cells was detected using an ROS assay kit. A549 cells grown in a logarithmic phase were inoculated in glass-bottomed culture dishes, and the number of cells was controlled at 5 × 10^5^ cells/mL. Lip-Cur, Lip-Ba and Lip-Cur/Ba solutions were prepared separately and diluted with cell culture medium to a drug concentration of 80 μg/mL, and incubated for 24 h. Then, the culture medium containing the drug was discarded and DCFH-DA was diluted with a serum-free culture medium at a ratio of 1:1000. Then, the culture medium containing the drug was discarded, DCFH-DA was diluted with the serum-free culture medium at a ratio of 1:1000 and 1 mL of DCFH-DA solution was added to each dish and incubated for 30 min [35], protected from the light. The solution was then discarded and washed with serum-free culture medium three times to remove the residual probes. Then, the probes were observed and photographed with a laser confocal microscope and recorded and the fluorescence intensity was measured using Image J. The fluorescence intensity of the probes was measured with a laser confocal microscope.

#### 2.2.13. In Vivo Antitumor Activity and Histological Analysis

Five-week-old male BALB/c mice were selected as animal models for the test and were housed in an SPF environmental system with a temperature of 20–26 °C, a humidity of 40–70%, circulating ventilation and light. Mice were acclimatized for 7 days prior to testing. A549 cells in a logarithmic growth phase were collected and digested using trypsin, the cell suspension was adjusted to 5 × 10^7^/mL and each mouse was injected subcutaneously with 0.1 mL of cell suspension. When the subcutaneous tumor grew to an obvious mass, the length (L) and width (W) of the tumor were measured daily and the volume was calculated (V = L × W^2^/2), and when the volume grew to 100 mm^3^ and the mouse’s weight was not significantly reduced, the administration of the drug was started.

All mice were randomly divided into the following two groups (*n* = 3): one group was injected with PBS solution and one group was injected with Lip-Cur/Ba (at a dose of 20 mg/kg). The administration method was intravenous and the frequency of administration was once every 2 days, for a total of seven times. The mice were terminated at the end of drug administration and the tumors were stripped off and weighed. The tumors were then soaked in formalin and sectioned by paraffin embedding. The sectioned tumor tissues were used for hematoxylin and eosin staining (H&E), terminal deoxynucleotidyl transferase-mediated dUTP nick-end labeling (TUNEL) and Ki67 staining, which were used to observe the structural and physiological changes of the cells within the tumor tissues, cell proliferation and apoptosis.

#### 2.2.14. Statistical Analysis

Data were analyzed by one-way analysis of variance (ANOVA) and Duncan’s multiple range test. Statistical analysis was performed with the software package SPSS^®^ 26.0 and *p* < 0.05 was deemed statistically significant.

## 3. Results

### 3.1. Morphology and Size of Lip-Cur/Ba

**Figure 1:**
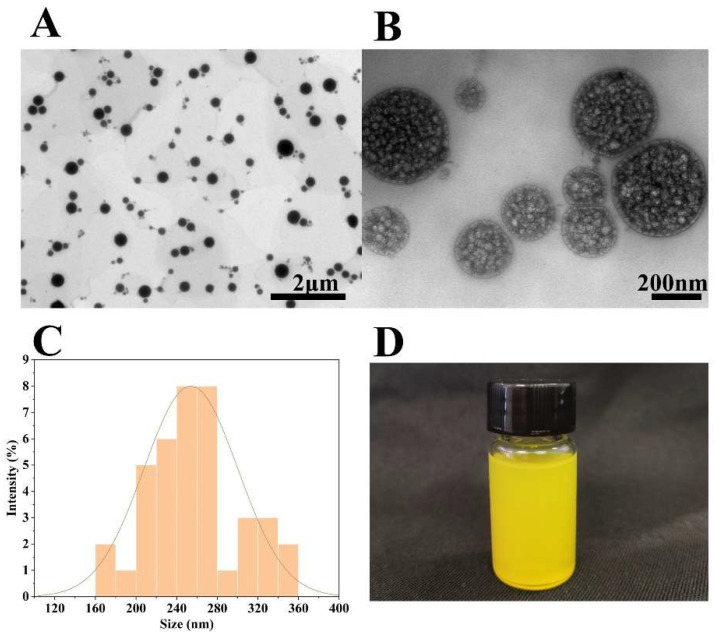
(**A**,**B**) TEM image of Lip-Cur/Ba at different magnifications; (**C**) the size of Lip-Cur/Ba; (**D**) appearance of Lip-Cur/Ba.

As shown in Figure 1A,B, which shows the images of Lip-Cur/Ba under a transmission electron microscope, it can be seen that Lip-Cur/Ba is mostly a regular round shape, and the magnified observation reveals that there are multiple vesicle structures within the Lip-Cur/Ba [36,37,38] and this structure can encapsulate more drugs to achieve a good encapsulation efficiency and loading capacity [39]. The samples of Lip-Cur/Ba were yellow, translucent and opalescent (Figure 1D). The average particle size measured by the particle sizer was 268 nm (Figure 1C), the zeta potential was −15.23 (mv) and the PDI was 0.104. Usually, the smaller the PDI is, the more homogeneous the molecular distribution in the system [40], which also proves that the prepared Lip-Cur/Ba has a certain stability [41].

### 3.2. Encapsulation Efficiency and Loading Capacity of Lip-Cur/Ba

In order to verify whether the B-Lip could successfully load two drugs, the concentrations of Cur and Ba were detected using UV spectrophotometry. According to the experimental results, the encapsulation efficiency of Cur in Lip-Cur/Ba was 97.23% and that of Ba was 94.06%, and the loading capacity of the whole system was 6.87%. This indicates that the B-Lip can be used as a drug-carrying system for Cur and Ba and can achieve good encapsulation efficiency and loading capacity of the drug, which can be continued for subsequent exploratory experiments. The encapsulation efficiency and loading capacity of Lip-Cur and Lip-Ba were also detected. The encapsulation efficiency of Lip-Cur was 86.45% and the loading capacity of Lip-Cur was 3.22%. The encapsulation efficiency of Lip-Ba was 68.52% and the loading capacity of Lip-Ba was 2.64%.

### 3.3. FT-IR Analysis

**Figure 2:**
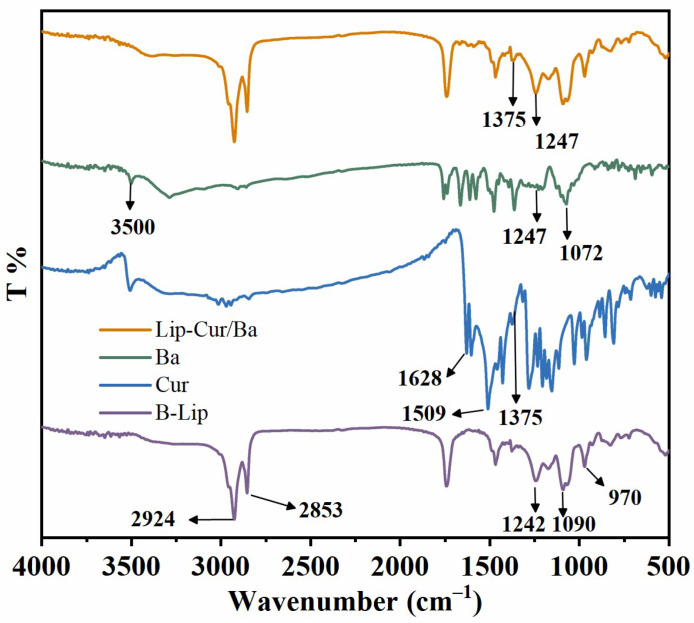
FT-IR spectra of B-Lip, Cur, Ba and Lip-Cur/Ba.

FT-IR spectroscopy is commonly used to analyze functional groups and structures in unknown substances. As shown in Figure 2, for the FT-IR spectroscopy of B-Lip, the absorption peaks at 2924 cm^−1^ and 2853 cm^−1^ are attributed to the stretching vibration of C-H in the low-end hydroxyl group of YL, the absorption peaks at 1242 cm^−1^ and 1090 cm^−1^ are attributed to the stretching vibration of the head group of Chol, PO^2−^ and the absorption peak at 970 cm^−1^ is attributed to the stretching vibration of N^+^-CH_3_ [42]. For the FT-IR spectroscopy of Cur, the absorption peak at 1628 cm^−1^ is attributed to the mixed vibration of C=O and C=C, the absorption peak at 1509 cm^−1^ is attributed to the telescopic vibration of C-O and C-C, the absorption peak at 1427 cm^−1^ is attributed to the bending vibration of the olefinic structure C-H and the absorption peak at 1375 cm^−1^ is attributed to the telescopic vibration of the C-O-C of the aromatic ring [43]. For the FT-IR spectroscopy of Ba, the absorption peak at 3500 cm^−1^ is attributed to the stretching vibration of the intermolecular hydroxyl group of baicalein, the absorption peaks at 1600–1700 cm^−1^ are attributed to the C=C and benzene ring C-H stretching vibration, the absorption peak at 1247 cm^−1^ is attributed to the stretching vibration of the benzene ring C=C and the absorption peak at 1072 cm^−1^ is attributed to the stretching vibration of the C-O-C [44]. Compared with the profiles of the above three substances, the FT-IR spectroscopy of Lip-Cur/Ba retained the characteristic peaks at 1375 cm^−1^ belonging to Cur and 1247 cm^−1^ to Ba, which indicated that Cur and Ba were successfully encapsulated into liposomes.

### 3.4. Thermogravimetric Analysis

**Figure 3:**
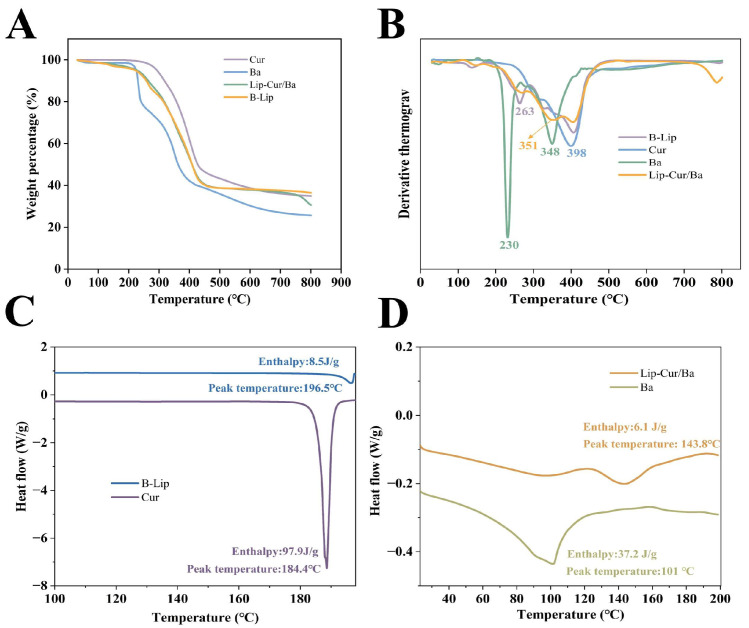
(**A**) TGA curves of Cur, Ba, Lip-Cur/Ba and B-Lip. (**B**) DTG curves of Cur, Ba, Lip-Cur/Ba and B-Lip. (**C**) DSC curves of B-Lip and Cur. (**D**) DSC curves of Lip-Cur/Ba and Ba.

As shown in Figure 3A,B, the thermal decomposition of B-Lip is concentrated between 220 °C and 420 °C, and the mass loss within this temperature is due to the loss of choline groups within the EYL [45]. The thermal decomposition of Cur occurs mainly at 398 °C. There are two loss platforms for Ba, and its thermal decomposition occurs mainly at 230 °C and 348 °C. Lip-Cur/Ba occurs mainly at 351 °C due to the weight loss peaks that occur as a result of the combined action of Cur and Ba, and the combined effect of EYL [46]. This indicates that Cur and Ba were successfully encapsulated into liposomes.

### 3.5. DSC Analysis

As shown in Figure 3C,D, the characteristic absorption peaks of B-Lip, Cur and Ba were 196 °C, 184 °C and 101 °C, and those of LIP-Cur/Ba were 94.19 °C and 143.83 °C. The characteristic peaks of Cur and Ba were not shown, which proved that the two drugs existed in liposomes in an amorphous form [47]. The lower enthalpy of absorption of 6.11 J/g for Lip-Cur/Ba compared to that of 8.52 J/g for B-LIP indicated that the drug was embedded in the hydrophobic inner layer of the phospholipid bilayer [48], which further demonstrated the successful encapsulation of the drug in liposomes.

### 3.6. In Vitro Releasing Assay Analysis

**Figure 4:**
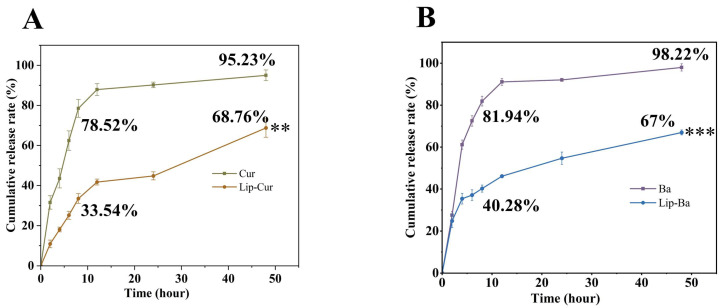
(**A**) Release behaviors of Lip-Cur and Cur (**: *p* < 0.01). (**B**) Release behaviors of Lip-Ba and Ba (***: *p* < 0.001).

As shown in Figure 4, the in vitro drug release profile of Lip-Cur/Ba shows the release behavior of Cur and Ba over a fixed period of time. For Cur and Ba, the control group had a fast release rate in the first 6 h phase, and the release had reached 78.52% and 81.94% in the first 8 h phase. The subsequent release rate gradually slowed down in the 12–48 h phase. Moreover, the drug was almost fully released in the first 48 h phase with a final release degree of 95.23% and 98.22%. For Cur and Ba in Lip-Cur/Ba, the release was only 33.54% and 40.28% at the first 8 h phase, and the release rate continued to increase steadily from 12 to 48 h. The final release was 68.76% and 67% at the first 48 h phase. This suggests that Lip-Cur/Ba has a slow release effect on the drug, thus prolonging the drug’s holding period, which is conducive to the utilization and efficacy of the drug in the treatment.

### 3.7. Cell Cytotoxicity of Lip-Cur/Ba

MTT can be reduced to formazan by succinate dehydrogenase in the mitochondria of living cells and DMSO can dissolve this water-insoluble blue-violet crystal and measure its absorbance at 490 nm with an enzyme marker. The number of living cells within a certain range is directly proportional to the magnitude of absorbance, which is able to respond to the proportion of living cells [49], thus inferring the cell-killing effect of Lip-Cur/Ba.

#### 3.7.1. Time-Dependence of Lip-Cur/Ba Action on A549

**Figure 5:**
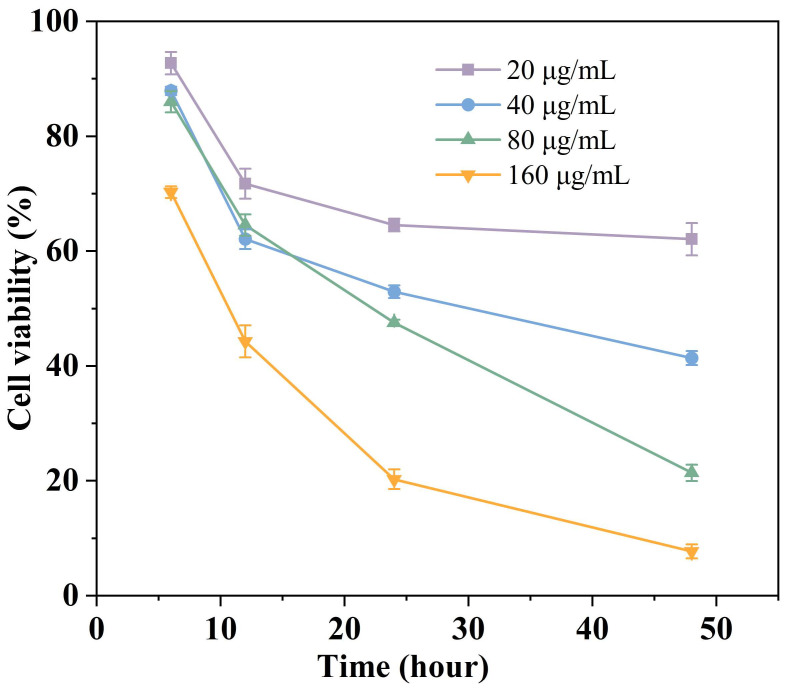
Time-dependence of Lip-Cur/Ba action on A549.

In order to investigate the inhibitory effect of Lip-Cur/Ba on A549 cells at different times and concentrations, different concentrations of Lip-Cur/Ba were co-cultured with A549 cells for 6, 12, 24 and 48 h, and the cell survival rate was determined by the MTT assay. As shown in Figure 5, the survival rate of the A549 cells showed concentration-time dependence. At the same time (6 h, 24 h, 48 h), the higher the concentration of Lip-Cur/Ba, the lower the survival rate of the A549 cells. At the same concentration, the cell viability gradually decreased with the increase in time. In all groups, the cell viability was still high at 6 h of co-culture, which was above 70%, and the inhibitory effect on the A549 cells was weak. When the co-culture time was delayed to 48 h, the survival rate of the Lip-Cur/Ba cells at a concentration of 160 μg/mL was only 7.74%, and the inhibitory effect on the A549 cells reached the maximum at this time, so that Lip-Cur/Ba was able to effectively eliminate hepatocellular carcinoma cells and exert the maximum efficacy of the drug. This result is consistent with the results of the previous in vitro release experiments, because the drug encapsulated in liposomes has the property of slow release, which results in less drug release in a short period of time, and the release of the drug has already reached more than 60% after 48 h, and thus can achieve the best drug effect. Based on the above results, 48 h of co-culture between the drug and the cells was chosen as the time point for the subsequent experiments.

#### 3.7.2. Cell Cytotoxicity of Lip-Cur/Ba on A549

**Figure 6:**
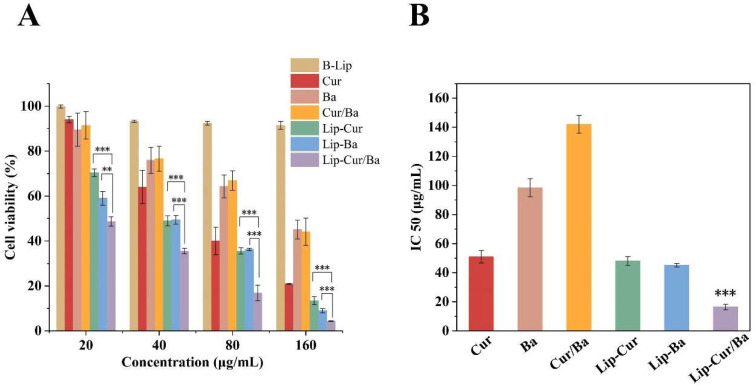
(**A**) Cell cytotoxicity of B-Lip, Cur, Ba, Cur/Ba, Lip-Cur, Lip-Ba and Lip-Cur/Ba. (**B**) IC50 of Cur, Ba, Cur/Ba, Lip-Cur, Lip-Ba and Lip-Cur/Ba (**: *p* < 0.01, ***: *p* < 0.001).

To investigate whether Cur and Ba co-embedded in liposomes were more effective in promoting apoptosis of A549, the cytotoxicity of B-Lip, Cur, Ba, Cur/Ba, Lip-Cur, Lip-Ba, Lip-Cur/Ba against A549 was evaluated using the MTT method. The IC50 values of each group and the combination index (CI) of the two drugs were also calculated. As shown in Figure 6A, the blank liposome B-Lip without drug loading had little toxic effect on cells at different concentrations, and the cell survival rates were all greater than 90%, which proved that the liposome carriers were safe and nontoxic for subsequent experiments. The cell survival rates of Cur and Ba dissolved with DMSO at the same concentration were significantly greater (*p* < 0.001) than those of Lip-Cur and Lip-Ba. Similarly, the cell survival rates of Cur/Ba dissolved and physically mixed with DMSO at the same concentration were greater than those of Lip-Cur/Ba, which suggests that the prepared B-Lip is effective for the encapsulation of drugs. In order to further determine whether Cur and Ba have a synergistic effect, this experiment also compared the cytotoxicity magnitude of dual-loaded and single-loaded liposomes, and it can be seen from Figure 6A that the cell survival rate of Lip-Cur/Ba was only 4.3%, which was significantly smaller (*p* < 0.001) than that of the Lip-Cur and Lip-Ba groups. In order to compare the effects of each group of drugs on cell survival more intuitively, the IC50 values of each group of experiments were calculated (Figure 6B), and it can be seen that the IC50 of free Cur, Ba and mixtures were greater than that of the liposome-loaded group, and that the IC50 value of Lip-Cur/Ba was 16.5 (μg/mL) and was significantly smaller than that of (*p* < 0.001) Lip-Cur and Lip-Ba IC50 values. According to the formula proposed by Chou and Talaay [50], the CI of the association index of Cur and Ba was calculated to be 0.5, when CI > 1 is antagonistic, CI = 1 is additive and CI < 1 is synergistic, and at this time, CI < 1 proves the synergistic effect of Cur and Ba. These results showed that the co-encapsulation of curcumin and baicalin into liposomes (Lip-Cur/Ba) were more effective in killing A549 than an individual agent (Lip-Cur and Lip-Ba), and verified the synergistic effect of the Cur/Ba combination.

### 3.8. Antimigration Capacity of Lip-Cur/Ba

Tumor cells can proliferate indefinitely in the body and have the ability to migrate, which leads to the proliferation and metastasis of cancer foci and is one of the most important reasons for the aggravation of cancer [51]. Therefore, analyzing the characteristics of cell migration has great significance to cancer research. Evaluation of antimigratory ability is also an important point in the evaluation of drug efficacy. We used the cell scratch assay and Transwell assay to evaluate the antilateral migration and antilongitudinal migration abilities of Lip-Cur/Ba.

#### 3.8.1. Results of Cell Scratching Experiments

**Figure 7:**
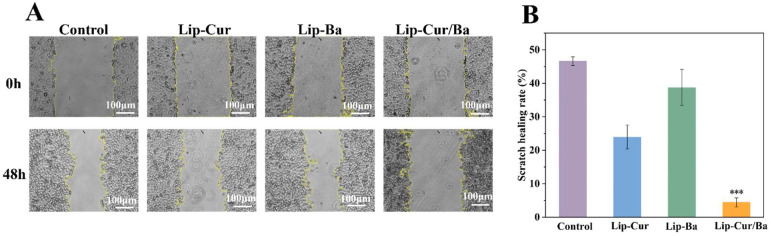
(**A**) Micrograph of the scratch experiments on A549 cells of Lip-Cur, Lip-Ba and Lip-Cur/Ba. (**B**) Scratch healing rate of Lip-Cur, Lip-Ba and Lip-Cur/Ba (***: *p* < 0.001).

In this experiment, a scratch was artificially created on the monolayer cells, and the cells at the edge of the scratch had a tendency to migrate to the blank area, thus simulating the environment of cell migration, and the area of the scratch was photographed and recorded at regular intervals so as to calculate the Lip-Cur/Ba anticell migration rate. As shown in Figure 7A, in the control group, the cells were not treated with any drugs, and after 48 h of incubation, the area of the cell scratches decreased and the borders appeared irregularly extending inward with new cells, which had an obvious tendency to migrate. The scratched area of cells treated with Lip-Cur, Lip-Ba and Lip-Cur/Ba in the experimental group was larger than that of the control group. And by quantifying the scratch area and cell migration rate, it can be found that the cell migration rate of Lip-Cur/Ba was only 4.43%, which was significantly smaller (*p* < 0.001) than that of the Lip-Cur and Lip-Ba groups. In addition, the healing rate of Lip-Cur/Ba was reduced by 91.3%, 82.6% and 89.5% compared with the control group, Lip-Cur group and Lip-Ba group (Figure 7B). This indicates that Lip-Cur/Ba can effectively inhibit the lateral migration of A549 and is more effective than single-carrier liposomes.

#### 3.8.2. Results of the Cell Transwell Assay

**Figure 8:**
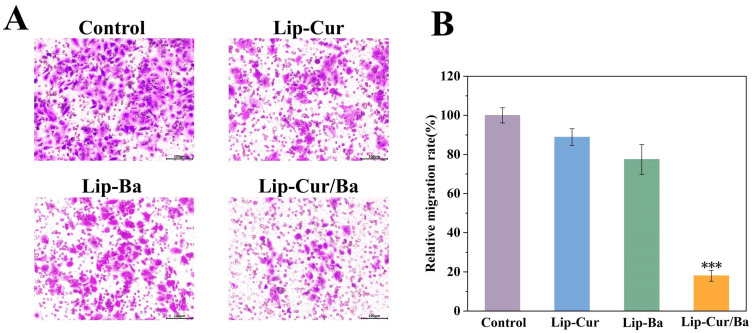
(**A**) Micrograph of the Transwell assay of Lip-Cur, Lip-Ba and Lip-Cur/Ba. (**B**) Relative migration rate of Lip-Cur, Lip-Ba and Lip-Cur/B (***: *p* < 0.001).

The Transwell chamber is divided into an upper chamber and a lower chamber with a polycarbonate membrane with small holes at the bottom of the upper chamber. Different culture solutions were added to the upper and lower chambers so that the effect of the components in the culture solution on cell movement and migration could be investigated. As shown in Figure 8A, the experimental group migrated significantly fewer cells into the lower chamber than the control group. The number of migrated cells was quantified using ImageJ. It can be found that the longitudinal migration rate of Lip-Cur/Ba was only 18.04%, which was significantly less (*p* < 0.001) than that of the Lip-Cur and Lip-Ba groups. Moreover, the migration rate of Lip-Cur/Ba was reduced by 82.06%, 79.53% and 76.62% compared with that of the control group, Lip-Cur group and Lip-Ba group (Figure 8B). This indicates that Lip-Cur/Ba can effectively inhibit the longitudinal migration of A549 and is more effective than single-carrier liposomes. Based on the above results, we can preliminarily deduce that Lip-Cur/Ba has a certain inhibitory effect on the migratory movement of A549 cells, and Lip-Cur/Ba has the best antimigration ability.

### 3.9. Results of ROS Level Detection

**Figure 9:**
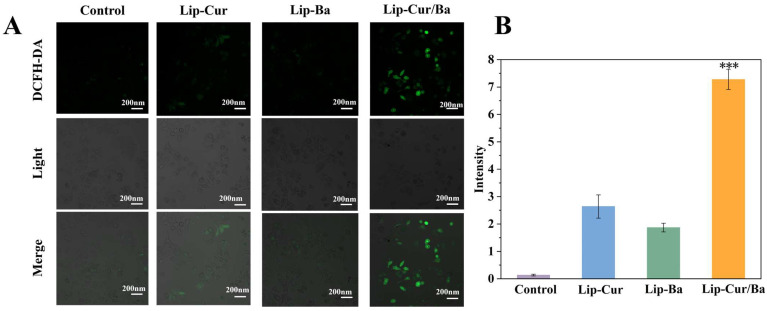
(**A**) Intracellular ROS levels photographed by an inverted-type laser scanning confocal microscope. (**B**) Quantitative analysis of intracellular ROS levels (***: *p* < 0.001).

Elevation of intracellular ROS can affect the viability status of cells and even lead to damage or apoptosis [52]. The DCFH-DA in the ROS assay kit used is nonfluorescent and can cross the cell membrane into the cell and be hydrolyzed by esterase to DCFH, whereas intracellular ROS can oxidize nonfluorescent DCFH to fluorescent DCF, and the stronger the fluorescent signal, the higher the intracellular ROS level. As shown in Figure 9A, in the control group without any drug treatment, the green fluorescence signal was almost invisible, which indicated that the cells in the control group were in a good state, the ROS level was very low and there was no apoptotic tendency. However, after the treatment of the liposome administration group, the green fluorescence signal could be observed to be enhanced, and the green fluorescence signal was the strongest in the Lip-Cur/Ba group. Therefore, we speculated that the liposome administration group might cause apoptosis by affecting the elevated intracellular ROS level, and the effect of the Lip-Cur/Ba group was significantly stronger (*p* < 0.001) than that of the Lip-Cur and Lip-Ba groups (Figure 9B).

### 3.10. In Vivo Antitumor Activity

**Figure 10:**
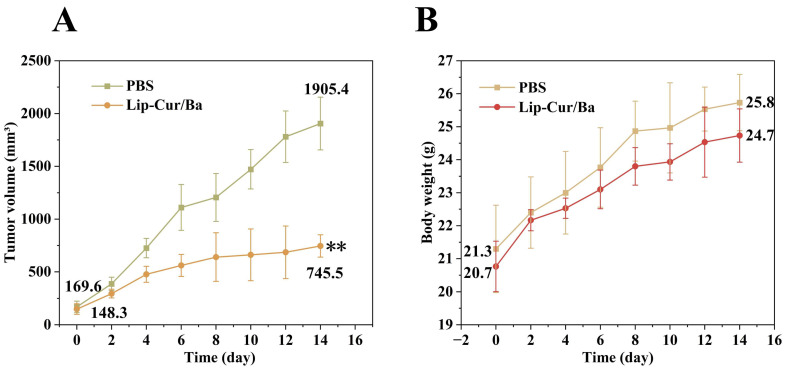
(**A**) Tumor volumes (mm^3^) and (**B**) body weights (g) of the A549 tumor-bearing mice in the different treatment groups (**: *p* < 0.01).

The antitumor activity of Lip-Cur/Ba was assessed by subcutaneous injection of A549 cells in BALB/c mice to form tumors to mimic the in vivo experimental setting. The results of in vitro experiments have demonstrated that Lip-Cur/Ba is significantly more potent than Lip-Cur and Lip-Ba in killing A549 cells, so the in vivo experiments mainly explored the antitumor activity of Lip-Cur/Ba. As shown in Figure 10A, which shows the change in tumor volume of each mouse, the tumors of control mice injected with PBS increased in volume over time, from only 169 mm^3^ on the first day at the start of the injections until the tumors had grown to 1905 mm^3^ on the 14th day at the end of the treatment, which is a more than 10-fold increase in volume. The tumor volume of Lip-Cur/Ba mice was 148 mm^3^ on the first day of the injections, and the subsequent growth rate was significantly inhibited, with a tumor volume of 745 mm^3^ at the end of 14 days, which was significantly smaller than that of the control group. After 14 days of treatment, the mice were terminated and the tumor tissues were stripped and weighed. The average mass of the tumors in the control group was 1629 g and that of the injected group was 485 g (Figure 10B), which proved that Lip-Cur/Ba had a certain inhibitory effect on the tumors. Moreover, the weight of the mice increased within 14 days of treatment, and there was no weight loss and other side effects, which proves the safety of Lip-Cur/Ba.

**Figure 11:**
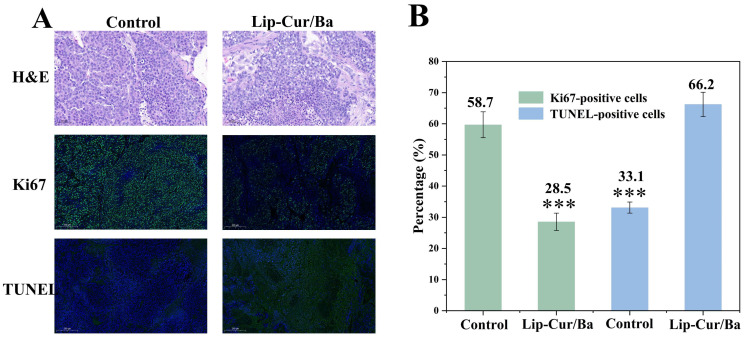
(**A**) Hematoxylin and eosin (H&E), Ki67 staining and terminal deoxynucleotidyl transferase-mediated dUTP nick-end labeling (TUNEL) of Lip-Cur/Ba tumor tissues on day 14. (**B**) Percentage of Ki67-positive and TUNEL-positive cells in tumor sections (***: *p* < 0.001).

To further confirm the therapeutic effect of Lip-Cur/Ba, tumor tissue was sectioned and examined. Hematoxylin and eosin (H&E) staining can reflect the structural and physiological changes of the cells in the tumor tissues, as shown in Figure 11A. In the sections of the control group, it can be seen that the tumor cells are arranged neatly and closely, the nuclei of the cells are in blue color and the majority of the cells are structurally intact, with regular shapes and no rupture phenomenon, from which it can be inferred that the tumor cells in the control group have a good growth status. In the sections injected with Lip-Cur/Ba, it can be seen that the tumor cells in the field of view are arranged in a crowded and disordered way, with inconsistent morphology and size, and there are obvious vacuoles in the cells and the nuclei of some cells are ruptured with no cytoplasm visible. This proved that under the action of Lip-Cur/Ba, the proliferation of tumor cells was obviously inhibited, and there was some necrosis.

The expression of Ki67 marks the level of tumor cell proliferation, as shown in Figure 11A. The expression of green signals was stronger in the control group and weaker and less in Lip-Cur/Ba, which proved that the proliferation of tumor cells was inhibited after the injection of Lip-Cur/Ba in mice. This was further confirmed in the TUNEL assay, where there was little green signal in the control group, indicating less apoptosis, whereas a large area of green fluorescence expression could be observed in Lip-Cur/Ba, proving that apoptosis was greatly increased. By analyzing the percentage of positive cells (Figure 11B), it can be seen that the percentage of Ki67-positive cells in Lip-Cur/Ba (28.54%) was significantly smaller than that in the control group (59.68%), and the percentage of TUNEL-positive cells in Lip-Cur/Ba (66.21%) was about twice as high as that in the control group (33.09%). These above results consistently demonstrated the inhibitory effect of Lip-Cur/Ba on tumor cells in vivo.

## 4. Discussion

Natural medical components show great potential in cancer treatment, whereas their poor solubility and low bioavailability limit their clinical application. In particular, some insoluble drugs such as curcumin and baicalin are difficult to absorb after direct oral administration or injection, and quickly degrade due to their instability [12,53]. Curcumin and baicalin have been found to suppress cancer progression and metastasis by blocking metalloprotease [54,55], and Cur/Bai-based combination therapy showed better anticancer effects than individual Cur application [20,21]. Hence, in the present study, the double-loaded nanoliposomes (Lip-Cur/Ba) have been developed for co-delivering curcumin and baicalin, aiming to overcome the application shortcomings. After preparation, optimization and characterization, the encapsulation efficiency of Cur in nanoliposomes was 97.23% and that of Ba was 94.06%, and the loading capacity of the whole system was 6.87%. In addition, Lip-Cur/Ba exhibited good physicochemical stabilities and sustained release property, as well as improved A549 cell inhibitory and antitumor activities. Moreover, in vitro and in vivo experiments verified the synergistic effect of the combination of curcumin and baicalin. In the following study, more in-depth research should be continued to explore the mechanism of the synergistic effect. Further chemical modification on the surface of liposome, such as functional groups and targeting substances, could be improved to achieve targeted delivery and to strengthen therapeutic effects. Additionally, the mass production method should be optimized for the wider application in clinical research.

## 5. Conclusions

In this study, we prepared a new drug-carrying system with a certain stability and slow-release function, Lip-Cur/Ba, which can effectively solve the drawbacks of curcumin and baicalin’s poor solubility and low bioavailability. The mean size, PDI and zeta potential of Lip-Cur/Ba were 268 nm, 0.104 and −15.23 mV. Meanwhile, the cytotoxicity test showed that Lip-Cur/Ba had good biosafety at the recommended dose. In addition, in vitro experiments showed that the co-encapsulated liposome of the two drugs has higher cytotoxicity and inhibition of cell migration against A549 cells, compared with the free-drug and single-drug carrier. Furthermore, in vivo experiments demonstrate the good antitumor activity of Lip-Cur/Ba in mice, which confirmed the strong inhibitory effect of Lip-Cur/Ba on non-small cell carcinoma. Moreover, these results verified the synergistic effect of the combination of curcumin and baicalin. In conclusion, Lip-Cur/Ba showed good therapeutic effects and potential research value on NSCLC, and this co-encapsulated liposome system should be applied in extended exploring.

## Data Availability

The data presented in this study are contained in the article.
